# Application of geographical information system (GIS) technology in the control of Buruli ulcer in Ghana

**DOI:** 10.1186/1471-2458-14-724

**Published:** 2014-07-16

**Authors:** Ernest Kenu, Vincent Ganu, Benedict NL Calys-Tagoe, Gerald AB Yiran, Margaret Lartey, Richard Adanu

**Affiliations:** 1 Korle-Bu Teaching Hospital, P.O.Box 77, Korle-Bu, Accra, Ghana; 2 Department of Geography and Resource Development, University of Ghana, Accra, Ghana; 3 University of Ghana Medical School, College of Health Sciences, Korle-Bu, Accra, Ghana; 4 University of Ghana, College of Health Sciences, School of Public Health, Accra, Ghana

**Keywords:** Buruli ulcer, Ghana, GIS, Mapping, Clustering, Case search

## Abstract

**Background:**

Buruli ulcer (BU) disease is a chronic debilitating skin disease caused by *Mycobacteriumulcerans.* It is associated with areas where the water is slow-flowing or stagnant. Policy makers take the necessary strategic and policy decisions especially where to target interventions based on available evidence including spatial distribution of the disease. Unfortunately, there is limited information on the spatial distribution of BU in Ghana. The aim of the study was to use Geographical Information System (GIS) technology to show the spatial distribution and hot spots of BU in Greater Accra and Eastern Regions in Ghana. The information could then be used by decision makers to make the necessary strategic and policy decisions, especially where to target intervention.

**Methods:**

We conducted a community case search and spatial mapping in two districts in Eastern region (Akuapem South and Suhum- Kraboa-Coaltar) and two districts in Greater Accra region (Ga West and Ga South Municipalities) of Ghana to identify the spatial distribution of BU cases in the communities along the Densu River. These municipalities are already known to the Ministry of Health as having high case load of BU. Structured questionnaires on demographic characteristics, environmental factors and general practices were administered to the cases.

Using the E-trex Garmin Geographical Positioning System (GPS), the location of the case patient was marked along with any important attributes of the community. ArcGIS was used to generate maps showing BU distribution and hot spots.

**Results:**

Two hundred and fifty-seven (257) probable BU patients were enrolled in the study after the case search. These cases and their houses (or homes) were located with the GPS. The GIS maps generated showed a varying distribution of BU in the various communities. We observed clustering of BU patients downstream of the Densu River which had hitherto not been observed.

**Conclusions:**

There is clustering of BU in areas where the river was most contaminated. The identified hot spots for BU should be targeted for interventions by policy makers to ensure effective control of BU in Ghana.

## Background

Buruli ulcer (BU) disease is a chronic debilitating skin disease caused by *Mycobacterium ulcerans*[1,2]. It is one of the neglected tropical diseases and second commonest disease caused by Mycobacterium in Ghana and third globally [3,4]. Currently, BU has been reported in over 30 countries, in the subtropical regions of Asia, Latin America, the Western Pacific region and Eastern and Central Africa [1,2,5,6]. Even though it has been reported in other continents, West Africa is the region most affected [1,6]. Bayley reported the first case of BU in Ghana in 1971 [7,8] and in 1999 there was national search for BU which found a prevalence rate of 87.7 per 100 000 population in Ga West District with the highest number of active cases [9]. Over 426 communities have reported cases of BU in Ghana. These communities are in Ashanti, Brong Ahafo, Eastern, Greater Accra and the Western regions of the country. Children less than fifteen years are mostly affected, even though persons of any age can be affected [6,10,11].

*Mycobacterium ulcerans* can be detected in both endemic and non-endemic sites although quantitative data is lacking for African countries [12]. It occurs in discrete foci suggesting a spatial correlate with infection. Due to the disparity between hospital and community data, a detailed small-scale study on the location of houses with BU with respect to specific features will lead to the identification of spatial correlates of infection.

Spatial epidemiology has proven to be useful for understanding the geographical distribution of many diseases [13-17]. Geographic Information Systems (GIS) technology offers the unique opportunity and the ability to collect vast amount of data over large spatial region and to make spatial analysis for identification of hotspots. Policy makers take the necessary strategic and policy decisions especially where to target interventions based on available evidence including spatial distribution of the disease. Unfortunately, there is limited information on the spatial distribution of BU in Ghana. The aim of the study was to use GIS technology to show the spatial distribution and hot spots of BU in Greater Accra and Eastern Regions.

## Methods

**Figure 1:**
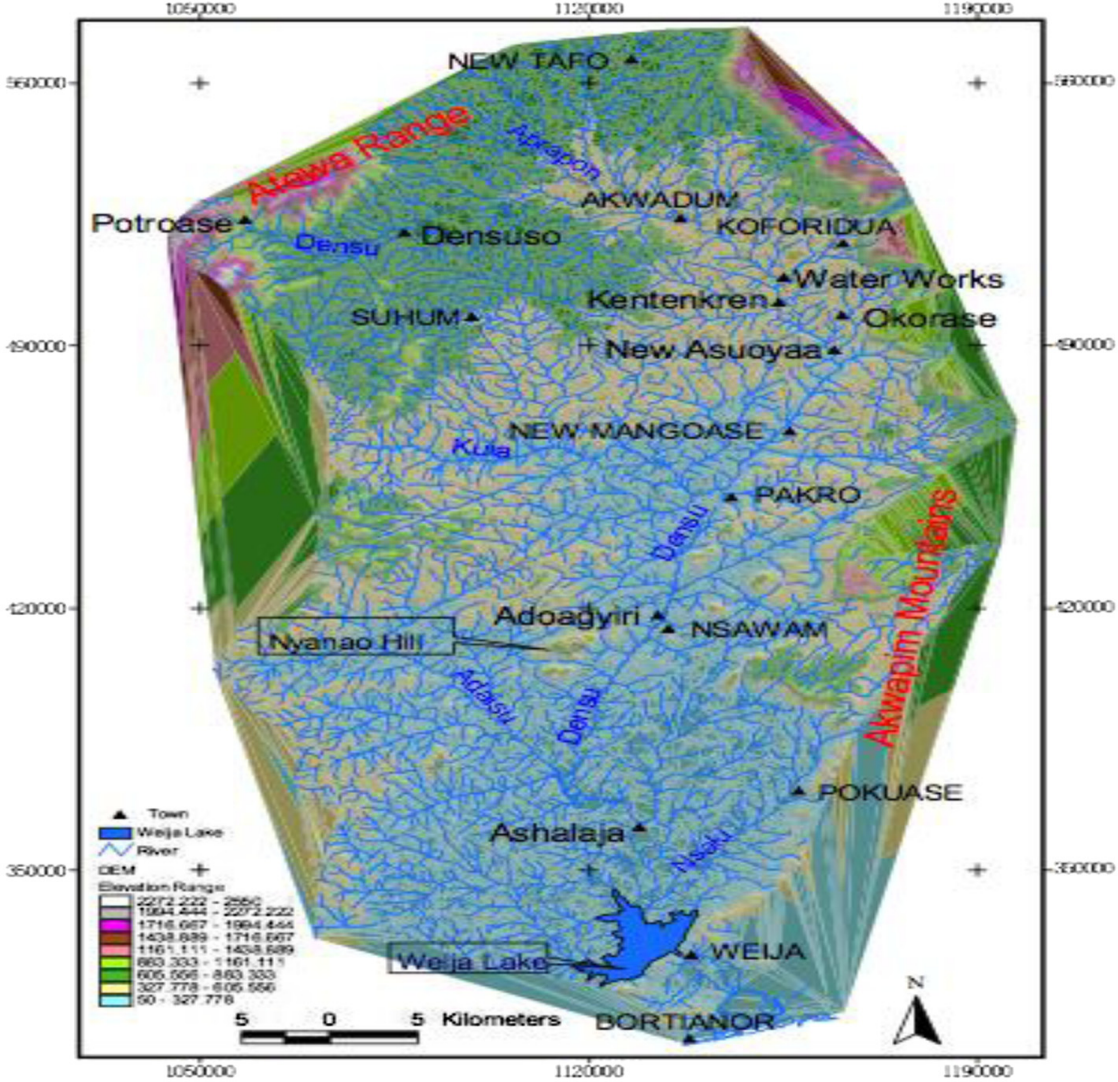
The basin of the Densu River (Source: Geography Department, University of Ghana).

An active community case search with mapping of BU cases in their respective houses along the entire course of the Densu River was carried out. The Densu River is 116 km long, and takes its source from the Atewa – Atwiredu Mountains near Kibi in the East Akyem District of the Eastern Region and enters the Gulf of Guinea at Bortianor, near Accra. It spans an area of about 2,490 km^2^. This area is surrounded by high mountains such as the Atewa range and the Akuapim Mountains thus making the area around the Densu relatively flat as shown in Figure 1. The water therefore flows fast at the sources on the mountains and slows down as it enters the Densu. This slow movement of the water towards the coast makes the area conducive for the breeding of the BU bacteria.

### Profile of study area

**Figure 2:**
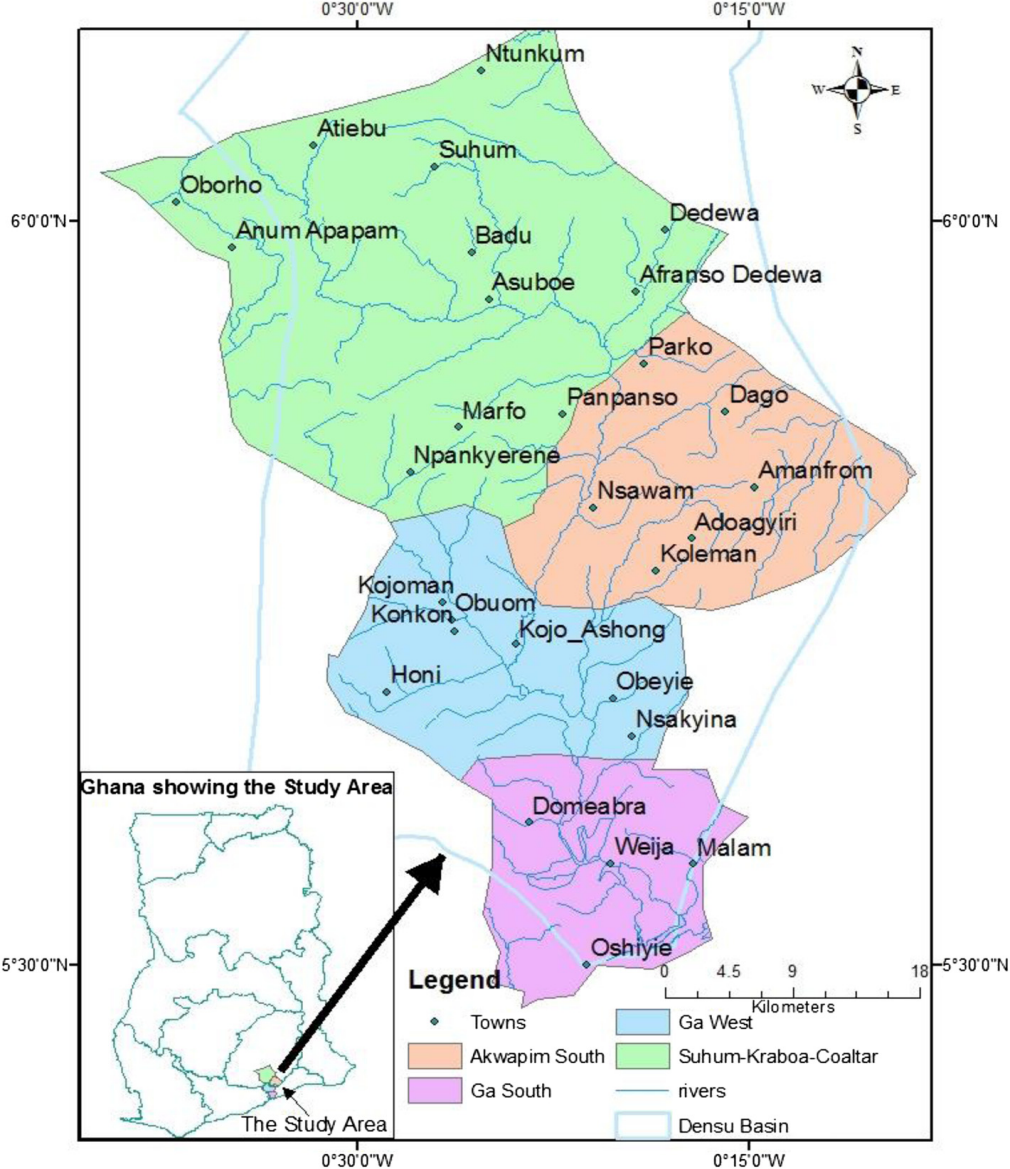
Map of the Densu River and the districts where the active case search was carried out.

The study was done in Akuapem South municipality, Suhum-Kraboa-Coaltar District, Ga West and South Municipalities (Figure 2). The populations of these districts as at the time of the study are shown in Table 1.

**Table 1:** Population of study districts

**District**	**Population**
Akuapem South Municipality	123,501
Suhum-Kraboa-Coaltar	167,551
Ga West Municipality	262,742
Ga South Municipality	485,643

Source: Ghana Statistical Service, 2010.

Ga West and South were originally one municipality and separated after the 2000 population and housing census. These districts are considered urban settlements with 60% of their population living in urban communities. The Akans, who are the indigenes, form the majority in Akuapem South and Suhum-Kraboa-Coaltar districts while the Ga-Adangmes are the majority in Ga West and South Municipalities. However, people from all walks of life are increasingly migrating into these districts. A large proportion of the inhabitants of these districts especially those in the rural areas draw their water for domestic use from rivers, ponds, dugouts wells and other open water sources that are most often contaminated [18]. The population of these districts is increasing and with it, the demand for safe and potable water. The increasing scarcity of potable water, forces more people to use unsafe water and thereby exposing themselves to water related disease such as BU.

### Study population

The target population for the study was made up of all people living in the four districts at the time of study.

### Case definition

Any person aged 2 years or more who resided in the Suhum-Kraboa-Coaltar, Akuapem South, Ga west and South districts diagnosed of BU meeting the WHO clinical case definition for *M. ulcerans* disease. WHO clinical case definition for BU divides the disease into two stages: active and inactive. The active form is characterized by non-ulcerative (papules, nodules, plaques, and edema) and ulcerative disease. The distinctive features of a BU include undermining edges, white cotton wool-like appearance, and thickening and darkening of the skin surrounding the lesion.

Clinically diagnosed BU patients were recruited into the study following an active case search in the communities by trained Community Based Surveillance Volunteers, Community Health Workers and principal investigators in all the four districts from May 2010 to December 2011. The study area was divided into geographic study areas to make numbering of the communities, active case search, detailed data collection and case tracing more manageable. Communities within the study area and 10 kilometers radius from the Densu River were included in the study.

The community active case search was done in three ways to ensure all the possible cases had been identified.

1. Use of trained Community Based Volunteers who had pictures of BU cases, skin conditions that could likely be BU and the case definition for BU

2. All children of School going age were thoroughly examined from head to toe for BU lesions

3. Self-referral through the use of the laminated pictures of BU lesions and WHO posters on BU posted in the various communities for awareness creation and easy identification of BU cases.

Samples were taken from all the active lesions for laboratory confirmation for positive polymerase chain reaction (PCR) or a positive Ziehl-Neelsen test for acid-fast bacilli in smears of lesions. The Laboratory confirmations were done at Noguchi Memorial Institute of Medical Research and all the positive cases were linked to care at any of the district/municipal hospitals closest to the patient eg Ga West Municipal Hospital, Kojo Ashong Health Centre, Pakro-Dago Health Centre and Nsawam Government Hospital.

E-trex Garmin Geographical positioning system (GPS) receiver was used to mark the location of each suspected BU patient. Houses, water bodies, field/crops, gardens, footpaths, rivers, roads and any other features of importance were also marked with the GPS receiver. In order to have accurate data from the Garmin eTrex Legend GPS receiver, the research assistants were trained on the use of the GPS receiver (DD, WGS-84, and WAAS-enabled). When the receiver had acquired signals from 4 or more satellites (3D fix), the coordinates were displayed on the screen and when the positional accuracy shown was less than or equal to 4meters (12.5 feet), then the point was taken. Accuracy Check Point (ACP) on the GPS receiver was set. Questionnaires administered collected information on demography (age, sex, place of residence, marital status, occupation, and educational status). GIS co-ordinates data taken for the cases were entered into an excel spread sheet and imported into ArcGIS software.

GIS maps were developed showing BU case distributions along the Densu River. Maps were generated based on BU cases per population of the communities and districts*.* Diameters called Buffer Zones were created around the places where BU cases reside to find out if there were particular environmental features there. Further analysis was done to determine the presence of clustering among the BU cases.

### Ethical approval

The Institutional Review Board (IRB) of Noguchi Memorial Institute for Medical Research and the Ethical Committee of Ghana Health Service gave ethical approval for the study. Permission was sought from community leaders, all adult subjects provided written informed consent and a parent or guardian of any child participant provided written informed consent on their behalf.

## Results and discussion

A total of 257 suspected buruli ulcer cases were identified from over 400 communities over a period of 18 months. Of the 257 BU cases, 85.6% (220/257) were active and the rest inactive. Ninety percent (198/220) of the active cases were pre-ulcers and 22 presented as ulcers. One hundred and eighty-seven (187) of the suspected cases were in Akuapem South, 22 in Suhum Kraboa Coaltar Districts, 40 in Ga west and 8 in Ga South Municipalities. Though not the first active case search for Buruli ulcer in Ghana [9], the unique nature of the planning, involvement of all stakeholders and linking the patients identified into care makes this study unique in the country.

**Figure 3:**
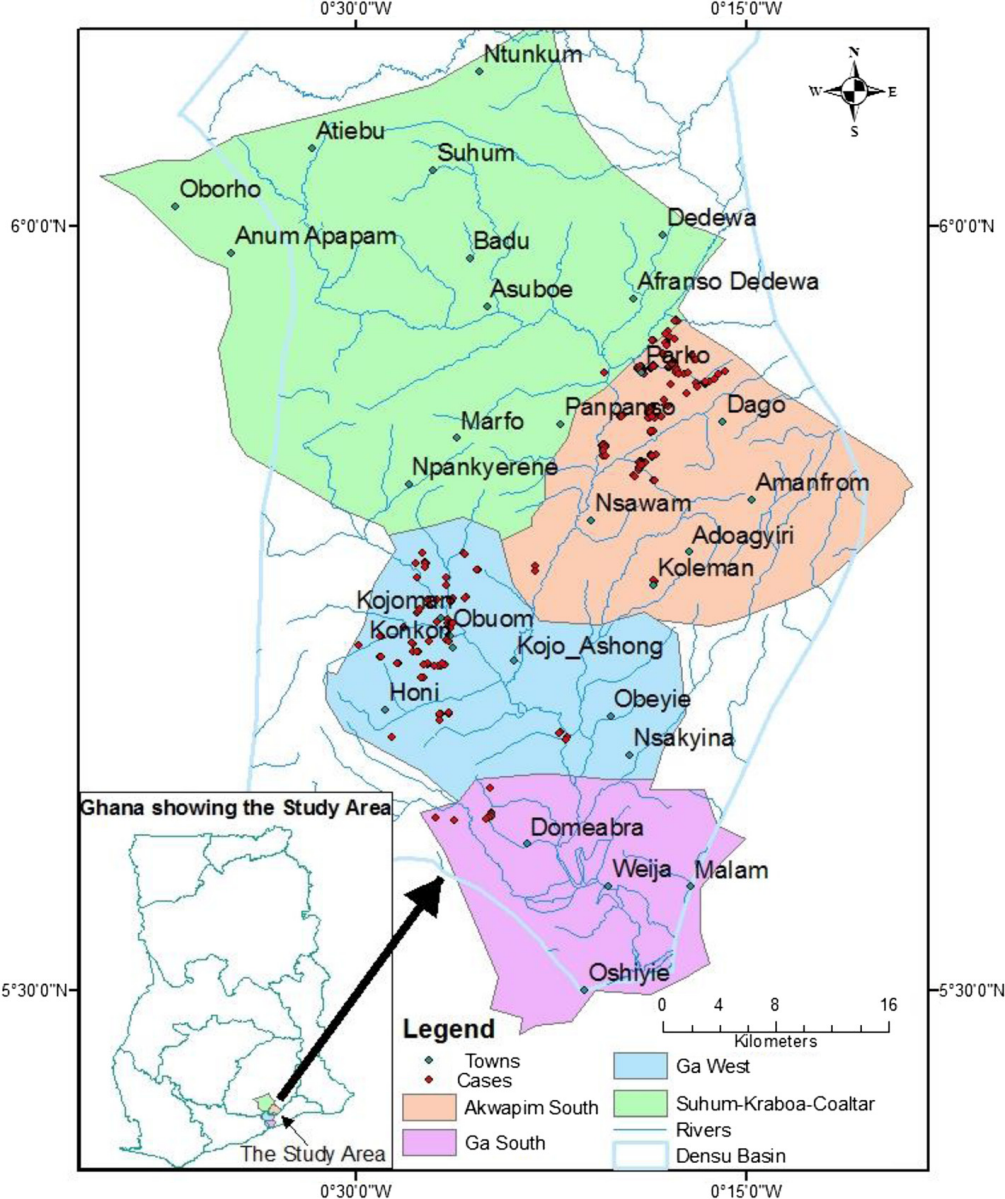
Map of study area with distribution of Buruli ulcer cases.

**Figure 4:**
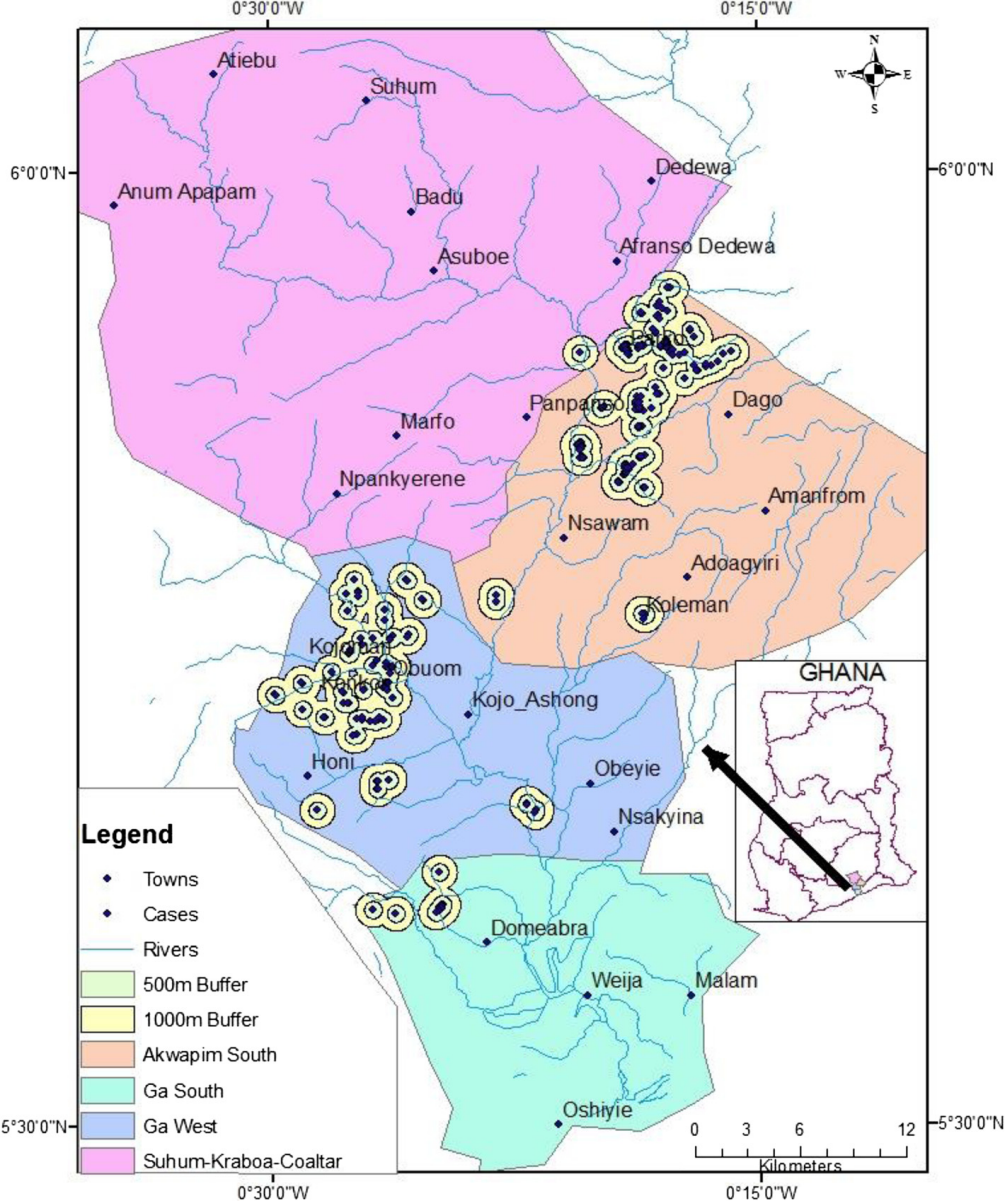
Distribution of Buruli ulcer cases with 500 and 1000 meters buffer.

**Figure 5:**
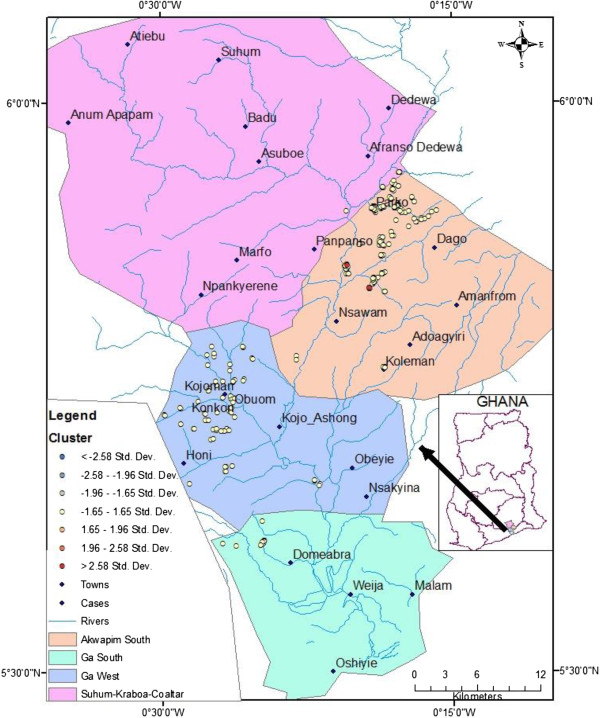
Distribution of Buruli ulcer cases with Clustering.

The hypothesis “There is no difference in the distribution of BU in the communities along the Densu river” has been clearly refuted with the mapping of the cases shown by Figures 3, 4 and 5. These figures show that the occurrence of BU was along the Densu river especially in areas where there are gentle slopes and the flow is very slow. A study done by Duker in Ghana in 2005 has also shown varying distribution of BU [19]. A similar approach has been used by other researchers to show where interventions were needed most [14,20-22]. In Koraput district in Orissa- India, a GIS based study was carried out for identification of risk factors based on ecological parameters for decision in support of formulation of appropriate control strategies for malaria [15]. Srivastava and team in 2009 used the same approach to identify malaria hot spots for focused intervention in tribal state of India. The study concluded that GIS mapping would make it easy to update information, identify hot spots at community levels within a district and this information can be graphically presented to policy makers to formulate focused and cost effective malaria control strategy [16]. The distribution of the cases were mainly in Akuapem south municipality and Ga West Municipalities. A buffer of 500 meters and 1000 meters were created around each case to look at the special features around the cases as shown in Figure 4. Most of the cases were in close proximity with water bodies as was demonstrated by Aiga and team in 2004. However, a large scale landscape-based model for predicting *Mycobacterium ulcerans* infection (Buruli ulcer Disease) presence in Benin found no association between percent of water land cover surrounding villages and the distance to the nearest river in relation to increased probability of BU presence at the village level [23]. The challenge with such a large scale study is its inability to have fine resolutions and therefore cannot take into account smaller water bodies evident at a small scale e.g. pond and wetlands. It looked at only major rivers and this could have influenced their results. In order to avoid this generalisation, this study considered all water bodies and was done at the local level.In Figure 5, clustering of BU cases was observed in Akuapem South Municipality and Ga West Municipality. This study demonstrated clustering of BU cases in Akuapem South District with Z-score > 2.58 which shows statistically significant hot spot for BU. Of the four districts where the study was performed the hottest spot was Akuapem South, followed by Ga West then Suhum-Kraboa-Coaltar and the least was Ga South.

**Figure 6:**
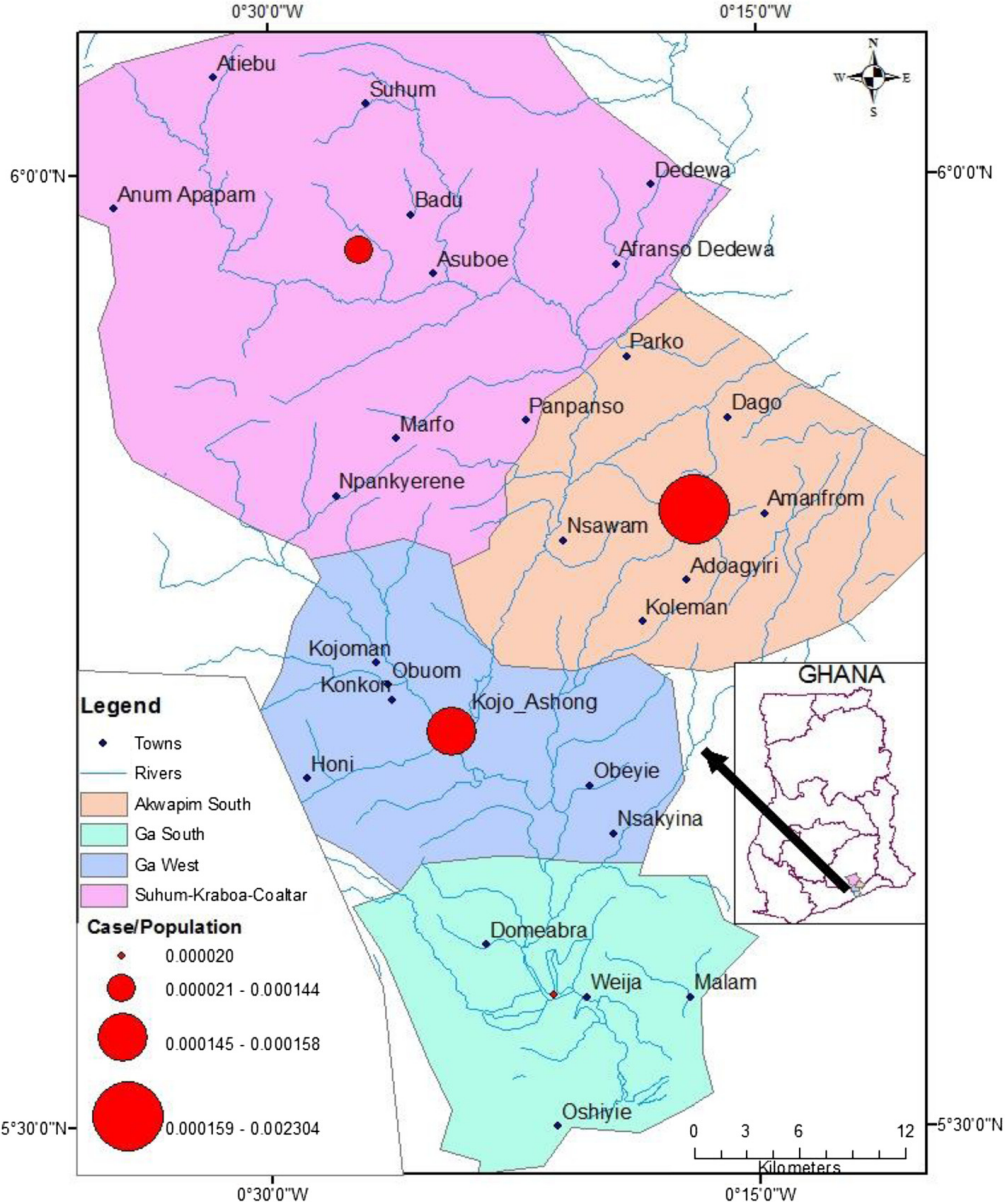
Distribution of Buruli ulcer Cases per population (Prevalence) of Districts.

The prevalence of BU for each district (Table 2) and risk level is shown in Figure 6. Though Ga West has been the district with the largest burden of Buruli ulcer over the years, this study has shown that the various interventions put in place in that district have been effective. This was especially evident in one of the reported highly endemic communities, Kojo Ashong, which had the lowest number of active Buruli ulcer cases. On the other hand areas like Akuapem South and Suhum-Kraboa-Coaltar which were not known to be endemic with Buruli ulcer are now becoming hot spots requiring urgent attention. This brings to light how GIS can help policy makers pictorially identify problem areas in order to use the information for strategic policy decisions for effective control and prevention of BU.

**Table 2:** District prevalence of Buruli ulcer

**District/Municipality**	**Population**	**Suspected cases**	**Prevalence/100,000 population**
Ga South	485,643	8	1.6
Ga West	262,742	40	15.2
Akuapem South	123,501	187	151.4
Suhum- Kraboa-Coaltar	167,551	22	13.1

## Conclusions

The goal of the study was to determine the spatial distribution of the disease in the communities along the Densu river. The GPS maps generated clearly show where the cases were coming from, the clustering nature of the disease and the risk level per population of the districts. In addition, this study has clearly shown that BU cases were not present upstream of the Densu river but rather were only seen from the point where the river was contaminated and flowed slowly.

## Competing interests

The authors declare that they have no competing interests.

## Authors’ contributions

EK Conceived and designed the study, carried out the research, involved in statistical analysis and drafted the manuscript. BNLCT participated in the design of the study, took part in the research and drafted the manuscript. VG was involved in the design of the study, carried out the research and drafted the manuscript. GABY helped designed the study, research field work, involved in statistical analysis and the realigned the drafted manuscript. ML Conceived and designed the study, carried out the research and drafted the manuscript. RA Conceived and designed the study, carried out the research and drafted the manuscript. All authors read and approved the final manuscript. Funding for this study was provided by the ACBRIDGE project.

## Pre-publication history

The pre-publication history for this paper can be accessed here:

http://www.biomedcentral.com/1471-2458/14/724/prepub
